# Linac-based stereotactic salvage reirradiation for intraprostatic prostate cancer recurrence: toxicity and outcomes

**DOI:** 10.1007/s00066-023-02043-3

**Published:** 2023-02-02

**Authors:** Salvatore Cozzi, Sebastiano Finocchi Ghersi, Lilia Bardoscia, Masoumeh Najafi, Gladys Blandino, Emanuele Alì, Matteo Augugliaro, Federica Vigo, Maria Paola Ruggieri, Raffaele Cardano, Lucia Giaccherini, Federico Iori, Andrea Botti, Valeria Trojani, Patrizia Ciammella, Cinzia Iotti

**Affiliations:** 1 Radiation Oncology Unit, Azienda USL-IRCCS di Reggio Emilia, 42123 Reggio Emilia, Italy; 2 Radiation Oncolgy Unit, AOU Sant’Andrea, Facoltà di Medicina e Psicologia, Università La Sapienza, 00185 Rome, Italy; 3 Radiation Oncology Unit, S. Luca Hospital, Healthcare Company Tuscany Nord Ovest, 55100 Lucca, Italy; 4 Skull Base Research Center, Iran University of Medical Science, 1997667665 Tehran, Iran; 5 Medical Physics Unit, Azienda USL-IRCCS di Reggio Emilia, 42123 Reggio Emilia, Italy; 6 Radiation Oncology Department, Centre Lèon Bèrard, Lyon, France

**Keywords:** Reirradiation, Prostate reirradiation, Volumetric modulated arc radiotherapy, Stereotactic body radiotherapy, Prostate local relapse

## Abstract

**Background:**

The rates of local failure after curative radiotherapy for prostate cancer (PC) remain high despite more accurate locoregional treatments available, with one third of patients experiencing biochemical failure and clinical relapse occurring in 30–47% of cases. Today, androgen deprivation therapy (ADT) is the treatment of choice in this setting, but with not negligible toxicity and low effects on local disease. Therefore, the treatment of intraprostatic PC recurrence represents a challenge for radiation oncologists. Prostate reirradiation (Re-I) might be a therapeutic possibility. We present our series of patients treated with salvage stereotactic Re‑I for intraprostatic recurrence of PC after radical radiotherapy, with the aim of evaluating feasibility and safety of linac-based prostate Re‑I.

**Materials and methods:**

We retrospectively evaluated toxicities and outcomes of patients who underwent salvage reirradiation using volumetric modulated arc therapy (VMAT) for intraprostatic PC recurrence. Inclusion criteria were age ≥ 18 years, histologically proven diagnosis of PC, salvage Re‑I for intraprostatic recurrence after primary radiotherapy for PC with curative intent, concurrent/adjuvant ADT with stereotactic body radiation therapy (SBRT) allowed, performance status ECOG 0–2, restaging choline/PSMA-PET/TC and prostate MRI after biochemical recurrence, and signed informed consent.

**Results:**

From January 2019 to April 2022, 20 patients were recruited. Median follow-up was 26.7 months (range 7–50). After SBRT, no patients were lost at follow-up and all are still alive. One- and 2‑year progression free survival (PFS) was 100% and 81.5%, respectively, while 2‑year biochemical progression-free survival (bFFS) was 88.9%. Four patients (20%) experienced locoregional lymph node progression and were treated with a further course of SBRT. Prostate reirradiation allowed the ADT start to be postponed for 12–39 months. Re‑I was well tolerated by all patients and none discontinued the treatment. No cases of ≥ G3 genitourinary (GU) or gastrointestinal (GI) toxicity were reported. Seven (35%) and 2 (10%) patients experienced acute G1 and G2 GU toxicity, respectively. Late GU toxicity was recorded in 10 (50%) patients, including 8 (40%) G1 and 2 (10%) G2. ADT-related side effects were found in 7 patients (hot flashes and asthenia).

**Conclusion:**

Linac-based SBRT is a safe technique for performing Re‑I for intraprostatic recurrence after primary curative radiotherapy for PC. Future prospective, randomized studies are desirable to better understand the effectiveness of reirradiation and the still open questions in this field.

## Introduction

Prostate cancer (PC) is one of the most common malignancies in the male population worldwide, with over 1 million new cases reported in 2018. In Italy, PC is currently the most frequent cancer diagnosis and accounts for 19% of all diagnosed cancers [1–3].

Primary definitive radiotherapy (RT) with or without concurrent and adjuvant androgen deprivation therapy (ADT) represents a milestone in the treatment of nonmetastatic, hormone-sensitive PC with curative intent. Despite advances in radiation treatment planning and delivery techniques, which have allowed more accurate locoregional treatments than in the past, the rates of local failure still remain high, with one third of patients experiencing biochemical failure and clinical relapse occurring in 30–47% of previously irradiated patients and in 38–54% postprostatectomy.

In this clinical scenario, the optimal management of intraprostatic recurrence after prior RT is still not standardized. Many therapeutic options are described in literature, i.e., salvage radical prostatectomy in selected cases [4], but with possible high local complication rates. Other local therapies, such as cryosurgery or high-intensity focused ultrasound (HIFU), could be considered, even if not reaching a wide consensus because of their possible adverse events, including fistula or severe rectal damage [5].

Currently, ADT is the treatment of choice in this setting, despite non-negligible systemic side effects and the probable development of castration resistance in the long term, together with little-proven benefits in terms of local control of disease [6]. Beyond these treatment modalities, reirradiation (Re-I) after local failure could be a therapeutic possibility. The critical issue of Re‑I is treatment tolerance of previously irradiated normal tissues and organs at risk (OARs) that could preclude dose delivery with curative intent [7]. However, taking into account the implementation of modern RT modalities, Re‑I has been considered as feasible for prostate cancer as for other solid tumors in clinical practice [8–11].

To date, prostate Re‑I appears to be the exclusive prerogative of very few cancer centers in the world.

The aim of the present work is to retrospectively evaluate our series of patients undergoing salvage reirradiation with a stereotactic technique for intraprostatic recurrence of PC after primary radical radiotherapy, with particular focus on toxicity outcomes and effectiveness.

## Materials and methods

We retrospectively evaluated toxicities and outcomes of patients with intraprostatic recurrence following primary radical radiotherapy for hormone-sensitive prostate-confined or locally advanced PC. Intraprostatic recurrent lesions were detected by 11C-/18F-choline-positron emission tomography/computed tomography (PET/CT) or 68Ga-prostate-specific membrane antigen (PSMA)-PET/CT after evidence of biochemical relapse (defined in accordance with the Phoenix criteria: PSA ≥ PSA nadir +2 ng/mL [12, 13]). All patients underwent subsequent 1.5 T multiparametric prostate magnetic resonance imaging (mpMRI) using T2-weighted (T2W), diffusion-weighted (DWI), and dynamic contrast-enhanced (DCE) for confirmation of intraprostatic recurrence identified on functional examination (PET). In case of concordance between PET and MRI, patients were considered eligible for reirradiation. We considered prostate biopsy not mandatory if all diagnostic findings were univocal in the presence of a body of evidence (PSA kinetics, prostate MRI, and/or PET-CT findings) in favor of local recurrence.

Salvage reirradiation was performed using volumetric modulated arc therapy (VMAT). The present study received final approval from the Institutional Ethical Committee (protocol code 140/2022/OSS/IRCCSRE, approved on 27/04/2022) and was performed in accordance with the principles of Good Clinical Practice (GCP) with respect of the ICH GCP guidelines and the ethical principles contained in the Helsinki declaration and its subsequent updates. A written consent form was obtained from each enrolled patient.

### Inclusion criteria

Inclusion criteria were age ≥ 18 years, histologically confirmed diagnosis of prostate cancer, patients who underwent salvage reirradiation for intraprostatic recurrence after primary radical radiotherapy for prostate cancer, concurrent/adjuvant ADT with stereotactic body radiation therapy (SBRT) was allowed, performance status ECOG 0–2, patients who underwent PET/CT and/or mpMRI for restaging after biochemical recurrence, signed informed consent.

### Exclusion criteria

Exclusion criteria were performance status ECOG 3 or worse, any psychologic condition affecting the possibility to sign informed consent, patients treated with systemic therapy (chemotherapy or abiraterone/enzalutamide), the presence of extraregional metastasis at the time of relapse, patients who did not undergo restaging PET/CT and/or mpMRI after biochemical recurrence.

### Radiotherapy treatment

For all patients, a simulation CT scan with 3‑mm slice thickness was performed in supine position using a Combifix immobilization system. Patients were required to have fixed (150 ml) bladder filling using a bladder catheter and an empty rectum (with enema 2 h before the procedure). Target volume delineation was performed using the ARIA® Oncology Treatment Planning System (TPS). The gross tumor volume (GTV) was outlined on the acquired CT scan. Image registration and fusion between the simulation CT scan and diagnostic mpMRI and PET/CT was performed to improve the accuracy of target volume delineation. The GTV corresponded to the visible lesion on mpMRI in case of a single intraprostatic lesion (18 patients). For bilateral recurrent lesions confirmed with MRI, the entire prostate was outlined as target volume (2 patients). A clinical target volume (CTV) was not created for stereotactic radiotherapy planning; therefore, the two volumes GTV and CTV coincided in our series. An isotropic 5–6-mm expansion was applied to the GTV/CTV to obtain the planning target volume (PTV). The outlined OARs were bladder, rectum, anterior and posterior rectal wall, femur heads, and penile bulb. The bladder catheter served as a surrogate for urethra contouring.

Radiotherapy planning was performed using VMAT with 6‑ or 10-MV flattening filter-free (FFF) beams. At least 95% of the PTV was required to receive 100% of the prescribed dose with a homogeneous distribution. Patient setup and the accuracy of target position were verified daily using cone-beam CT. A consecutive five-fraction SBRT regimen for a total target dose of 30 Gy was applied, administered every other day.

Acute toxicity was evaluated and graded according to the National Cancer Institute Common Terminology Criteria for Adverse Events v.5.0 (CTCAE). The normal tissue dose constraints published by Jereczek-Fossa et al. [14] and D’Agostino et al. [15] were used for treatment planning and are summarized in Table 1.

**Table 1:** Normal tissue dose constraints

Bladder	Urethra	Rectum	Rectal wall
D30 < 10 Gy	Dmax < 120%	D30 < 13.5 Gy	Dmax < 100%
V12 < 15%	V50105%	V18 < 20%	–
V10 < 20%	V24 < 30%	V10 < 40%	–
–	–	Dmax < 40 Gy	–

*D* Dose, *V* volume, *Dmax* maximum dose

### Androgen deprivation therapy

The addition or omission of ADT was evaluated in each individual case and depended on the characteristics of the primary tumor (in particular, additional ADT was preferred for primary high-risk or locally advanced disease) and the time interval between primary treatment and local recurrence (additional ADT preferred in patients with a short-interval recurrence). ADT consisted of LH-RH analog drugs, concomitant and adjuvant to SBRT, administered for 12 months. The use of ADT was excluded in the presence of severe cardiological comorbidities.

### Follow-up

The follow-up schedule consisted of clinical examination (to evaluate toxicity) and PSA detection every 3 months for the first year after Re‑I, then every 6 months. Restaging of disease with CT scan plus bone scan or functional imaging (11C-choline-PET/CT or 68Ga-PSMA PET/CT) was performed in patients with rising PSA (using the Phoenix criteria and with a focus on the PSA doubling time) and/or new-onset symptoms (urinary retention, hematuria, or bone pain refractory to pain relief).

Any symptom reported within 3 months of the end of reirradiation was considered acute toxicity; manifestation of symptoms 6 months after the end of treatment was considered late toxicity.

### Definition of clinical outcome

The following parameters were used to evaluate the clinical outcomes: overall survival (OS): time between the end of reirradiation treatment and patient’s death from any cause; progression-free survival (PFS): time between the end of reirradiation treatment and the date of disease recurrence and/or progression, taking into account the sum of the events “treatment failure”; biochemical failure: defined in accordance with the Phoenix criteria (PSA ≥ PSA nadir +2 ng/mL); and finally, biochemical progression-free survival (bPFS) was defined as the time between the end of reirradiation treatment and +biochemical recurrence.

## Results

From January 2019 to April 2022, 20 patients met the inclusion criteria. Median follow-up was 26.7 months (range 7–50). Median age was 78 years (range 56–82). Median time from primary RT to Re‑I was 73.8 months (range 21–146).

### Characteristics of the population in primary treatment

The characteristics of the population in the study are summarized in Table 2. According to the D’Amico risk classification, 5 (25%) patients had low-risk PC, 7 (35%) intermediate-, and 8 (40%) high-risk disease at presentation. Patients were distributed in the following way regarding the ISUP grade group (GG): GG 1: 5 (25%); GG 2: 4 (20); GG 3: 8 (40); and GG 4: (15). All but one patient underwent conventionally or moderately hypofractionated radiotherapy as primary radical treatment, which was given as primary SBRT for a total dose of 36.25 Gy in 5 fractions (without pelvic irradiation). Half of the patients received pelvic irradiation, the other half were treated only to the prostate gland.

**Table 2:** Patients and tumor baseline characteristics

Characteristic	Value (%)
**Primary treatment**	
*Whole population*	20
*Median follow-up*	26.7 months (range 7–50)
*Median age*	78 years (range 56–82)
*T stage*	
T1	7 (35)
T2	6 (30)
T3	6 (30)
T4	1 (5)
*ISUP grade group (GG)*	
1	5 (25)
2	4 (20)
3	8 (40)
4	3 (15)
*N stage*	
N0	15 (75)
NX	2 (10)
N1	3 (15)
*Risk group at diagnosis*^*a*^	
Low	5 (25)
Intermediate	7 (35)
High	8 (40)
*Median dose (primary treatment)*	70 Gy (range 35–78.2)
*Median no. fractions (primary treatment)*	25 (range 5–39)
*Pelvic irradiation*	
Yes	10 (50)
No	10 (50)
*Primary ADT*	
Yes	9 (45)
No	11 (55)
**Reirradiation**	
*Staging*	
Choline-PET	17 (85)
PSMA-PET	3 (15)
*Median PTV size (cc)*	13.6 (range 7.2–76)
*Concurrent Re‑I and ADT*	
Yes	13 (65)
No	7 (35)

*ISUP* international Society of Urological Pathology, *N* lymph node, *ADT* androgen deprivation therapy, *PTV* planning target volume, *Re‑I* reirradiation

^a^Risk group according to D’Amico classification

### Characteristics of the population in reirradiation

**Fig. 1:**
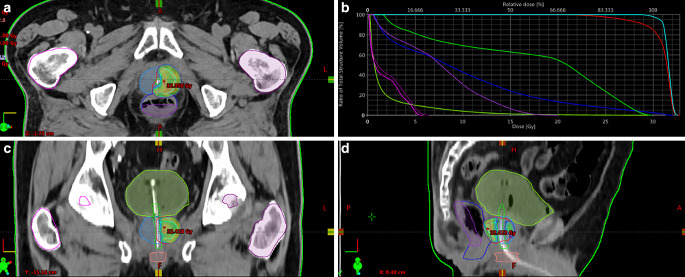
Example of planning with axial (**a**), coronal (**c**) and sagittal (**d**) dose distribution and dose volume histogram (**b**)

Median PTV size was 13.6 cc (range 7.2–76). Reirradiation on the entire prostate was performed in 2 patients (10%) with bilateral intraglandular disease recurrence, while the target volume was represented by the single macroscopic lesion highlighted in PET/CT and mpMRI in the remaining cases (Fig. 1).

**Fig. 2:**
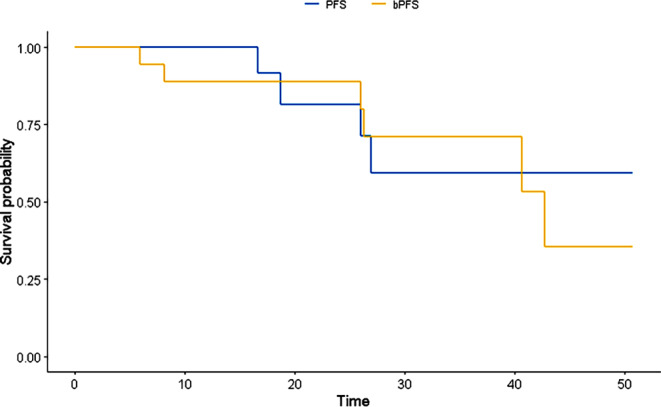
Progression-free survival (*PFS*; *blue line*) and cumulative biochemical progression-free survival (*bPFS*; *yellow line*)

**Fig. 3:**
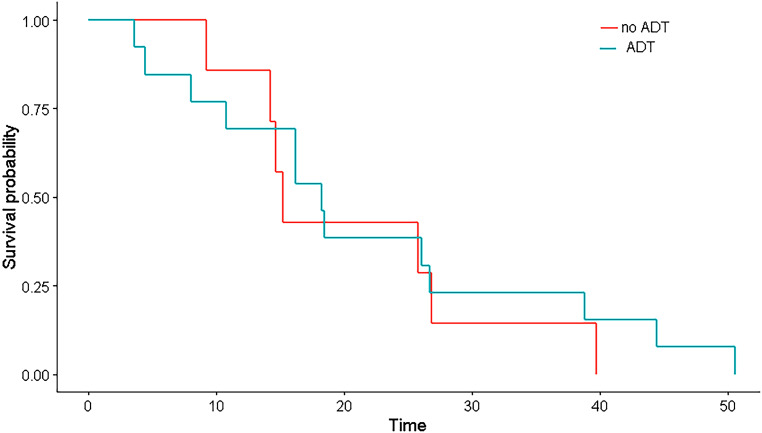
Biochemical progression-free survival in two populations: with concurrent androgen deprivation therapy (*ADT*; *blue line*) and without ADT (*red line*)

After SBRT Re‑I, no patients were lost to follow-up and all were alive at the time of the analysis. Two-year overall survival (OS) was 100%, 1‑ and 2‑year progression-free survival (PFS) were 100% and 81.5%, respectively, while 2‑year biochemical progression-free survival (bPFS) was 88.9%. Two-year local control of disease was 100%: no further intraprostatic relapse was recorded. Survival curves are showed in Fig. 2. Concurrent and adjuvant ADT with SBRT was administered in 9 patients (45%) and bPFS was evaluated by separating patients treated with and without ADT: 1–2-year bPFS without ADT was 71.4% and 28.6%, respectively, while with ADT was 69.2% and 3.8%, respectively (Fig. 3).

As regards PSA, 17 patients had a reduction in the PSA value 3 months after reirradiation, while 3 patients did not have a response in terms of PSA reduction, with an increase at the first follow-up. In all 3 cases, concomitant hormone therapy was administered, while all patients treated without concomitant hormone therapy had an initial drop in PSA.

Four patients (20%) experienced locoregional lymph node disease progression documented in PSMA-PET and were treated with a new course of SBRT. Two patients developed progression of disease 12 months after stereotactic Re‑I, the remaining two 24 months after SBRT. Biochemical failure but no pathological uptake areas on restaging PET/CT were reported in 1 (5%) patient; therefore, he continued follow-up with quarterly PSA detection. In patients who had not received concomitant hormone therapy, a postponement of ADT start by 12 to 39 months was also recorded with the use of prostate Re‑I. Moreover, all patients who received hormone therapy maintained, at the end of hormone therapy, a PSA value below the biochemical relapse value for more than 6 months from the last follow-up.

### Toxicity

Prostate Re‑I was well tolerated by all patients any treatment interruptions were reported. Table 3 summarizes the main toxicity recorded. No ≥ G3 genitourinary (GU) or gastrointestinal (GI) acute toxicities were reported. Only 1 (5%) patient experienced G1 diarrhea. Seven (35%) and 2 (10%) patients experienced acute G1 and G2 GU toxicity, respectively. Pollakiuria, dysuria, and stranguria were the main recorded symptoms. The symptoms lasted on average 2 weeks for G2 side effects and resolved with administration of local and systemic steroid therapy. Late GU toxicity was recorded in 10 (50%) patients, including 8 (40%) G1 and 2 (10%) G2. There was no late GI toxicity of any grade. Finally, ADT-related toxicity was found with the appearance of hot flashes and asthenia.

**Table 3:** Toxicity outcomes

Toxicity grade	Acute GI	Acute GU	Other acute toxicity	Late GI	Late GU	Other late toxicity
G1	1 (5%)	7 (35%)	Asthenia 5 (25%) Hot flashes 6 (30%)	0	8 (40%)	Asthenia 2 (10%) Hot flashes 4 (20%)
G2	0	2 (10%)	0	0	2 (10%)	0
G3	0	0	0	0	0	0
G4	0	0	0	0	0	0
G5	0	0	0	0	0	0

*GI* gastrointestinal, *GU* genitourinary

### Outcomes and toxicities

Reirradiation was well tolerated in all treated patients. Toxicities and outcomes are summarized in Table 2.

## Discussion

Radiotherapy plays a crucial role in the management of PC with curative intent as well as in the salvage setting. Emerging data have established RT as a useful therapeutic option also for oligometastatic and oligorecurrent/oligoprogressive disease [16], rare histologies [17, 18], or in combination with new drugs available for hormone-sensitive and castration-resistant PC. In recent years, advances in radiation planning and delivery techniques, in particular the advent of new-generation linac with the FFF mode, have improved treatment accuracy and given rise to the adoption of ultra-hypofractionated radiation schedules in the form of SBRT [19] in different oncological settings, with acceptable toxicity [20–25].

Despite this, local failure after RT still remains a critical issue. In this setting, ADT still represents the standard therapeutic approach, with a well-known, quite scarce, and time-limited benefit, and some unavoidable consequences on the patient’s quality of life. These patients could still benefit from a local treatment with the aim of achieving local control of disease and possibly postponing the need for systemic therapies [26, 27]. Salvage prostatectomy may offer a chance of cure, although it is burdened with important sequelae such as anastomotic stricture, urinary incontinence, and rectal injury. Conversely, HIFU has been associated with lower local control rates and a higher incidence of toxicities [28]. In this scenario, prostate Re‑I represents a challenge. Some authors have speculated that disease relapse after RT may be related to radioresistant tumor cell clones; therefore, a second course of radiation may not achieve a good oncological outcome. On the other hand, local tumor control has been postulated to eliminate a possible source of metastatic spread [29], so it may be hypothesized that targeting local cancer relapse may improve the prognosis of advanced malignancies [30].

Currently, salvage prostate Re‑I is the prerogative of few centers with high-volume experience. A recent systematic review by the Reirradiation Study Group of the Italian Association of Radiation Oncology (AIRO) showed that salvage brachytherapy is the most commonly used radiation technique for post-EBRT intraprostatic tumor recurrence [31]. Brachytherapy allows delivery of high doses to the target volume with remarkable OAR sparing in view of a rapid dose fall-off outside the sources, and more and more experiences have been published on this [32–51], so that it is recommended by NCCN guidelines, too. Some other reports describe prostate stereotactic Re‑I using the Cyber Knife [52–57], or external beam Re‑I with VMAT [14, 15, 58, 59]. According to the aforementioned latest systematic review of the literature [31], 18 articles are available in the current literature concerning salvage prostate Re‑I, accounting for a total of 511 patients with a median follow-up of 22 months (range 9.6–77.6). However, more recent articles have been published in the last 2 years [60–68].

In our series, the median follow-up was 26.7 months and the median time from primary RT to Re‑I was 73.8 months. Two-year OS and PFS were 100% and 81.5%, respectively, while 2‑year (bPFS) was 88.9%. We also found 100% local disease control and all the recruited patients were free from local failure at the time of the analysis. One- and 2‑year bPFS without ADT were 71.4% and 28.6%, respectively, while with ADT these values were 69.2% and 3.8%, respectively. We recorded only 4/20 cases of clinical progression of disease, all with nodal involvement documented by 68Ga-PSMA PET/CT and treated with a new course of ablative SBRT [16]. Unfortunately, the relatively short follow-up period and the relatively small sample size make it difficult to compare our data with those reported in literature. Nevertheless, the use of SBRT with FFF mode is relatively new in the setting of prostate Re‑I. A few studies have reported arguable survival outcomes, with 2‑year bPFS of 40–73% and 2‑year local control between 58% and 75% [31, 69].

Of note, in our series, prostate Re‑I was associated with very low toxicity rates, in line with the available literature. In fact, no ≥ G3 GU toxicity was recorded, and only 10% of the patients experienced acute G2 and late G2 GU side effects as the maximum grade of reported toxicity. Moreover, none of our patients experienced acute or late GI toxicity. Nearly one third of patients complained of ADT-related symptoms, such as hot flashes and fatigue as the most commonly described acute adverse event. Munoz et al. [31] reported a pooled result of acute ≥ G3 GU toxicity of 1.4% (95% CI 0.7–3%) and late ≥ G3 GU toxicity of 8.7% (95% CI: 5.8–13%) from 29 series, while no acute or late ≥ G3 GI toxicities occurred in the majority of the analyzed studies. In our experience, such low toxicity rates could be attributable to some of the following tools: first, SBRT performed every other day. Second, the relatively small PTV size obtained with the help of pretreatment mpMRI for target volume delineation. A pilot study by Sardaro and colleagues in 10 patients with postprostatectomy recurrent PC recently showed significantly lower mpMRI-based clinical target volumes than CT-based RT planning (*p* = 0.0003), with better OAR sparing and contemporary nonhomogeneous dose distribution, leading to an eventually aggressive dose escalation to the GTV [70]. The better soft tissue contrast provided by MRI and the advent of functional MR sequences may improve the definition of the prostate boundaries and pelvic OAR anatomy, the precise location of intraprostatic lesions, and thus the accuracy and safety of ablative, high-precision radiation treatments with linac, even in the setting of reirradiation [71–73]. Third, the use of a bladder catheter ensured high reproducibility of the treatment and excellent urethral sparing, and turned out to be fundamental to preventing obstructive and irritative urinary complications together with anti-inflammatory premedication with low doses of corticosteroids (prednisone 25 mg) concurrent to SBRT. Many authors have speculated that low toxicity rates may also depend on a long time interval between primary RT and Re‑I, while no author has demonstrated a statistical correlation between toxicity outcomes and dosimetric parameters. Some authors have correlated Re‑I of the entire prostate gland with higher toxicity rates. However, there was no increased GU or GI toxicity in the 2 patients with whole-prostate Re‑I in our series. Conversely, Zilli et al. reported severe adverse events and poor oncological outcomes using prostate Re‑I in a series of 14 patients treated with conventional or moderate hypofractionation plus brachytherapy or EBRT boost [74], and concluded that “Reirradiation on whole-gland EBRT with or without BT boost as salvage option may result in a relatively poor long-term outcome with a fairly high rate of severe side effects,” although most of the patients were treated with 3D radiotherapy in the absence of image-guided radiotherapy (IGRT).

Not less importantly, local treatments may be an attractive therapeutic strategy to postpone the need for ADT and its unavoidable long-term complications ranging from QoL impairment to metabolic changes and related systemic adverse events and, finally, the onset of tumor castration resistance [75]. The role of ADT concomitant to Re‑I is still controversial, since a clear correlation with cancer prognosis has not yet been demonstrated. Our findings showed that 45% of the patients did not receive concurrent or adjuvant ADT, while salvage prostate Re‑I allowed the start of palliative ADT to be delayed by up to 3 years. Moreover, all patients who received hormone therapy maintained, at the end of hormone therapy, a PSA below the biochemical relapse value for more than 6 months from the last follow-up, demonstrating a good biochemical response of SBRT treatment also in this patient setting.

It might be assumed a potential synergistic effect between local SBRT and systemic ADT. The su of ADT can also useful to obtain a prostate volume reduction and consequently better dose distribution [54, 76]. On contrary, the consensus advice of the Delphi group was not to administer ADT during Re‑I, probably because the advantage could be twofold, i.e., postponing an effective systemic treatment option and avoiding its potential side effects [77]. Fuller et al. [52] documented ADT-free survival (ADT-FS) as a clinical outcome, with a 5-year rate of 69%. In line with this, despite a shorter follow-up interval and a smaller sample size, a 2-year ADT-FS rate of 75% was reported by Cuccia et al. [78].

### Study limitations

The main limitation of our study was its retrospective design. In addition, the small sample size significantly reduced the opportunity to identify clear risk factors and efficacy predictors related to the presented treatment approach, although few studies with a similar number of cases have been reported in literature. The relatively short follow-up time (26.7 months) must also be considered, which did not permit accurate assessment of long-term toxicity outcomes. Important variability in the characteristics of the patients during first treatment is recorded (for example, the irradiation of the pelvis, the hormonal therapy, and the presence of some patients with lymph node disease), and this could influence the outcome of the patients analyzed. Finally, ADT was administered, according to the clinical characteristics of the disease, for a duration ranging from 6 to 12 months; therefore, it was not possible to precisely define its impact on outcomes. Moreover, no patient had a rebiopsy at the time of recurrence, and therefore the diagnosis of intraprostatic recurrence was guided by PET and subsequently confirmed by MRI. The absence of a rebiopsy did not allow us to reevaluate any modification of the histological characteristics of the primary tumor at the time of recurrence. Finally, it should be noted that in our series, an intrafractional control was not used, which is often strongly suggested in this treatment setting.

## Conclusion

Our experience supports the use of linac-based SBRT as a salvage reirradiation technique for intraprostatic recurrence after primary EBRT for prostate cancer as a feasible and well-tolerated treatment option with minimal toxicity. Image registration with pretreatment mpMRI and use of a bladder catheter and anti-inflammatory steroid premedication make salvage ultra-hypofractionated Re‑I with linac-FFF mode cost effective and extremely accurate. Our findings need confirmation in wider series with long-term follow-up, and randomized prospective trials are desirable.
